# Analysis of SARS-CoV-2 RNA Persistence across Indoor Surface Materials Reveals Best Practices for Environmental Monitoring Programs

**DOI:** 10.1128/mSystems.01136-21

**Published:** 2021-11-02

**Authors:** Rodolfo A. Salido, Victor J. Cantú, Alex E. Clark, Sandra L. Leibel, Anahid Foroughishafiei, Anushka Saha, Abbas Hakim, Alhakam Nouri, Alma L. Lastrella, Anelizze Castro-Martínez, Ashley Plascencia, Bhavika K. Kapadia, Bing Xia, Christopher A. Ruiz, Clarisse A. Marotz, Daniel Maunder, Elijah S. Lawrence, Elizabeth W. Smoot, Emily Eisner, Evelyn S. Crescini, Laura Kohn, Lizbeth Franco Vargas, Marisol Chacón, Maryann Betty, Michal Machnicki, Min Yi Wu, Nathan A. Baer, Pedro Belda-Ferre, Peter De Hoff, Phoebe Seaver, R. Tyler Ostrander, Rebecca Tsai, Shashank Sathe, Stefan Aigner, Sydney C. Morgan, Toan T. Ngo, Tom Barber, Willi Cheung, Aaron F. Carlin, Gene W. Yeo, Louise C. Laurent, Rebecca Fielding-Miller, Rob Knight

**Affiliations:** a Department of Bioengineering, University of California San Diego, La Jolla, California, USA; b Division of Infectious Diseases and Global Public Health, Department of Medicine, University of California San Diego School of Medicine, La Jolla, California, USA; c Department of Pediatrics, University of California San Diego, La Jolla, California, USA; d Sanford Consortium of Regenerative Medicine, University of California San Diego, La Jolla, California, USA; e Expedited COVID Identification Environment (EXCITE) Laboratory, Department of Pediatrics, University of California San Diego, La Jolla, California, USA; f Herbert Wertheim School of Public Health, University of California, La Jolla, California, USA; g Rady Children’s Hospital, San Diego, California, USA; h Department of Obstetrics, Gynecology, and Reproductive Sciences, University of California San Diego, La Jolla, California, USA; i Department of Cellular and Molecular Medicine, University of California San Diego, La Jolla, California, USA; j San Diego State University, San Diego, California, USA; k Department of Computer Science and Engineering, University of California San Diego, La Jolla, California, USA; l Center for Microbiome Innovation, Jacobs School of Engineering, University of California San Diego, La Jolla, California, USA; Tufts University

**Keywords:** COVID-19, RT-qPCR, SARS-CoV-2, environmental monitoring, heat-inactivated, surface sampling, swab

## Abstract

Environmental monitoring in public spaces can be used to identify surfaces contaminated by persons with coronavirus disease 2019 (COVID-19) and inform appropriate infection mitigation responses. Research groups have reported detection of severe acute respiratory syndrome coronavirus 2 (SARS-CoV-2) on surfaces days or weeks after the virus has been deposited, making it difficult to estimate when an infected individual may have shed virus onto a SARS-CoV-2-positive surface, which in turn complicates the process of establishing effective quarantine measures. In this study, we determined that reverse transcription-quantitative PCR (RT-qPCR) detection of viral RNA from heat-inactivated particles experiences minimal decay over 7 days of monitoring on eight out of nine surfaces tested. The properties of the studied surfaces result in RT-qPCR signatures that can be segregated into two material categories, rough and smooth, where smooth surfaces have a lower limit of detection. RT-qPCR signal intensity (average quantification cycle [*Cq*]) can be correlated with surface viral load using only one linear regression model per material category. The same experiment was performed with untreated viral particles on one surface from each category, with essentially identical results. The stability of RT-qPCR viral signal demonstrates the need to clean monitored surfaces after sampling to establish temporal resolution. Additionally, these findings can be used to minimize the number of materials and time points tested and allow for the use of heat-inactivated viral particles when optimizing environmental monitoring methods.

**IMPORTANCE** Environmental monitoring is an important tool for public health surveillance, particularly in settings with low rates of diagnostic testing. Time between sampling public environments, such as hospitals or schools, and notifying stakeholders of the results should be minimal, allowing decisions to be made toward containing outbreaks of coronavirus disease 2019 (COVID-19). The Safer At School Early Alert program (SASEA) (https://saseasystem.org/), a large-scale environmental monitoring effort in elementary school and child care settings, has processed >13,000 surface samples for SARS-CoV-2, detecting viral signals from 574 samples. However, consecutive detection events necessitated the present study to establish appropriate response practices around persistent viral signals on classroom surfaces. Other research groups and clinical labs developing environmental monitoring methods may need to establish their own correlation between RT-qPCR results and viral load, but this work provides evidence justifying simplified experimental designs, like reduced testing materials and the use of heat-inactivated viral particles.

## OBSERVATION

Development and characterization of methods for environmental monitoring of severe acute respiratory syndrome coronavirus 2 (SARS-CoV-2) remain important areas of research for identifying and mitigating potential outbreaks as the global pandemic continues. Environmental monitoring offers indirect detection of possibly infectious individuals through noninvasive sampling. In spaces with relatively consistent occupants, detection of SARS-CoV-2 from environmental samples can help identify coronavirus disease 2019 (COVID-19)-infected individuals, ideally before further transmission. Environmental monitoring can also alert public health leadership to the potential presence of an infection even in settings with low diagnostic testing uptake, allowing for the implementation of enhanced nonpharmaceutical interventions (i.e., double masking, increased hand hygiene, improved ventilation efforts) even in the absence of positive diagnostic tests.

SARS-CoV-2 particles are shed by symptomatic and asymptomatic carriers (1) and have been detected on various surfaces (2–5). Viral signatures have been demonstrated to persist up to 4 weeks in bulk floor dust collected from a room with a quarantined individual (6). Previous environmental monitoring studies have detected SARS-CoV-2 on surfaces contaminated by infected individuals in hospitals and congregate care facilities (6–10). Thus, indoor surface sampling can be valuable for detection of infected persons indoors, where transmission risk is highest (11). The Safer At School Early Alert program (SASEA) (https://saseasystem.org/) uses environmental monitoring and collected over 13,000 surface swabs, but we need more information to clarify what these data are telling us over time.

We sought to characterize temporal dynamics underlying detection of SARS-CoV-2 signals from surface swabs from a variety of common indoor surface types using reverse transcription-quantitative PCR (RT-qPCR). The Centers for Disease Control and Prevention (CDC) maintains that the risk of fomite transmission of SARS-CoV-2 is low (12). This study makes no claims of attempting to understand the possibility of or mechanisms behind infection of virus transmitted by fomites but rather on whether and how negative and positive RT-qPCR detection from surface swabs can enable decision-making in outbreak mitigation, focused clinical testing of individuals, and safe reopening of high-traffic, public spaces.

We used RT-qPCR to detect heat-inactivated viral particles on nine surface materials and monitored the persistence of the heat-inactivated virus for 7 days. Each material—acrylic, steel, glass, ceramic tile, melamine-finished particleboard (MFP), painted drywall, vinyl flooring, and two different carpets (olefin and polyester)—was divided into 5-cm by 5-cm grids, and each 25-cm^2^ square surface of the grid was inoculated with 10 μl of either a dilution series of heat-inactivated SARS-CoV-2 particles or water. The eight-point dilution series was based on viral genomic equivalents (GEs) as measured by digital droplet PCR (ddPCR). The inoculum dried for 1 h before swabbing. Every 24 h postinoculation, an unswabbed section of each material grid was sampled, for a total of 7 days, including the initial postinoculation swab.

To determine whether use of heat-inactivated viral particles in testing and validating environmental monitoring methods reflects results obtained using untreated virus, we compared detection of heat-inactivated SARS-CoV-2 (strain WA-1, SA-WA1/2020) and of authentic, untreated SARS-CoV-2 (variant of concern Beta, isolate B.1.351, hCoV-19/USA/MD-HP01542/2021) on two materials under biosafety level 3 (BSL-3) conditions.

### Findings.

**FIG 1:**
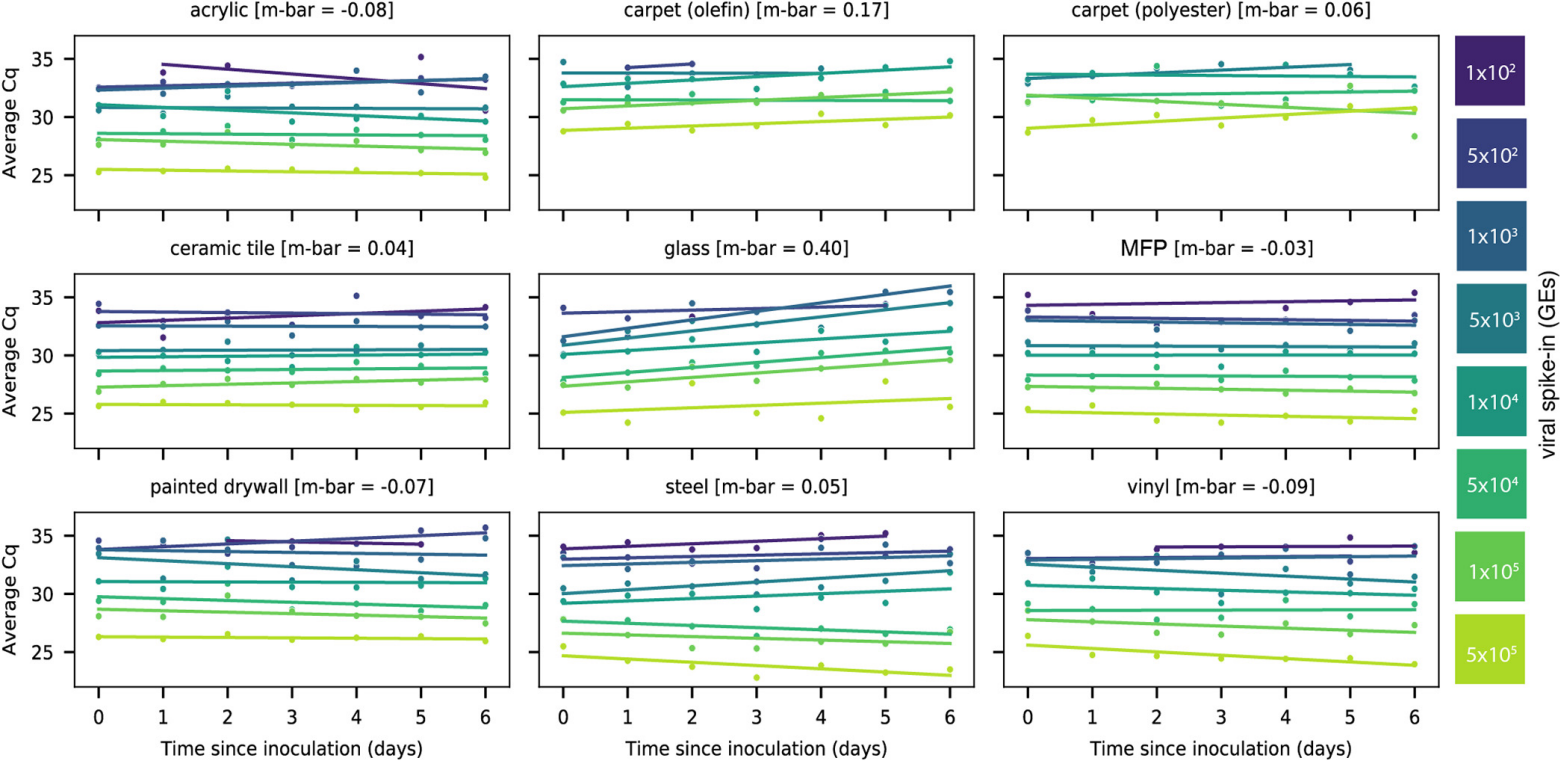
Scatterplots showing the average *Cq* values of RT-qPCR viral gene calls for corresponding heat-inactivated viral spike-in over 7 days. Viral spike-in concentrations reported as GEs from ddPCR. Linear regressions of average *Cq* values on days since inoculation per spike-in were overlaid on the measured data. The average decay slope (m-bar) is reported alongside each surface type.

Linear regression of signal intensity (average *Cq* of viral gene calls) on elapsed time since inoculation (days) for each dilution showed minimal decay of viral RNA on eight of nine surface types over 6 days (Fig. 1). The average decay slope for each surface type (m-bar) did not differ significantly from zero (mean = 0.0407, standard deviation [SD] =0.210). RT-qPCR signal decayed with time only on glass (m-bar = 0.401, SD =0.212, differing from the population mean by >1.5 standard deviations).

**FIG 2:**
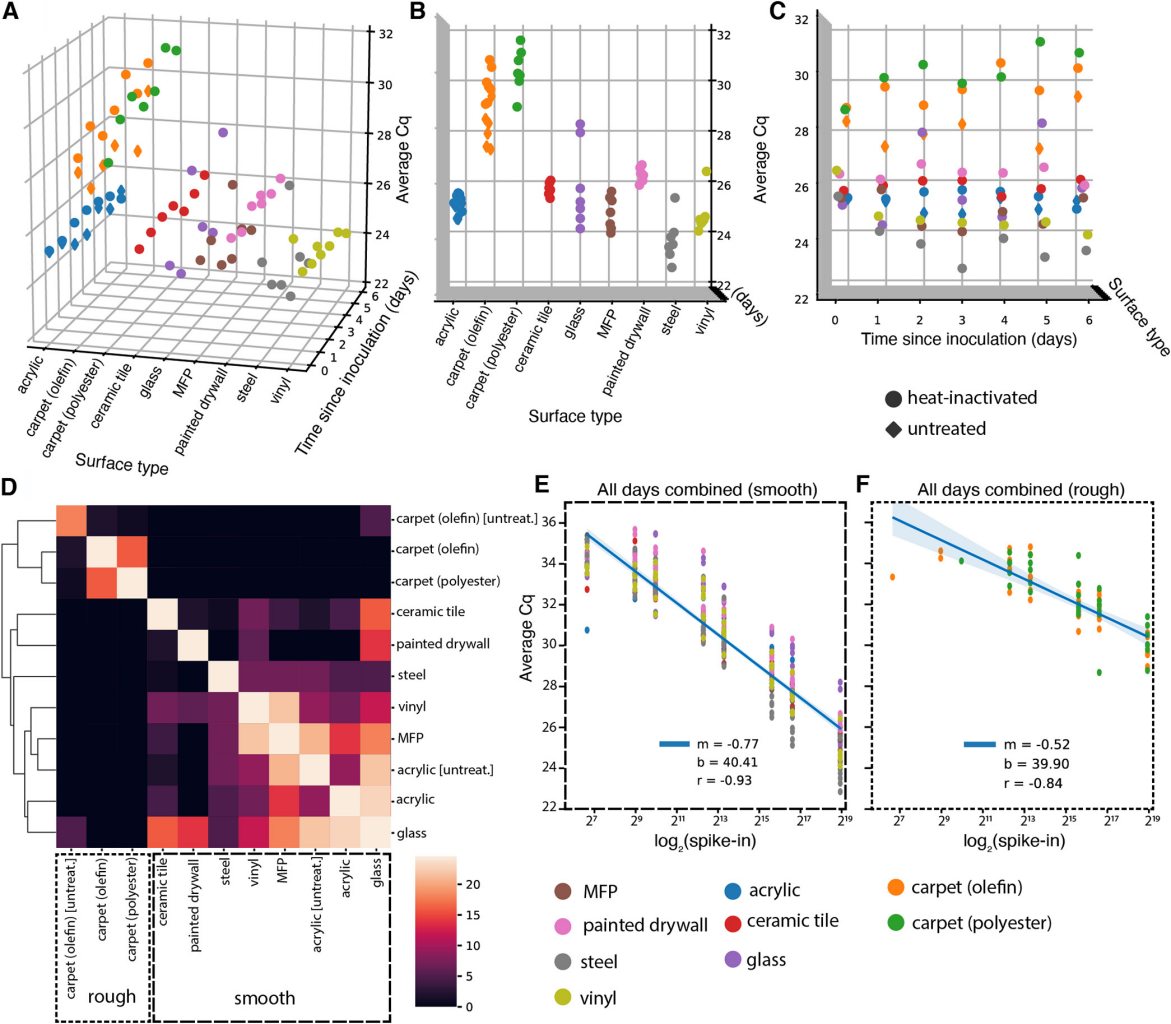
(A to C) 3D scatterplots showing distribution of average *Cq* values of viral gene calls over 7 days for nine different surfaces inoculated with 5 × 10^5^ GEs (nine surfaces for heat-inactivated virus [circles], two surfaces [acrylic and olefin carpet] for infectious virus [diamonds]). The distribution of *Cq*s differs significantly across surface types (B), but not across days since inoculation (C). (D) Clustermap of the *U* statistic from pairwise Mann-Whitney *U* tests between surface types. (E and F) Standard curves relating surface viral load (log_2_ spike-in) to average *Cq* values across all time points for smooth (E) and rough (F) surface types.

A two-way repeated measure analysis of variance (ANOVA) on viral signal intensity (average *Cq*) revealed that surface type explains more observed variation in *Cq* than does time since inoculation at the highest concentration (5 × 10^5^ GEs) (Fig. 2A). A Kruskal-Wallis *H* test confirmed that mean *Cq*s differ significantly across surface types (H = 60.86, *P* = 2.49 × 10^−9^) (Fig. 2B), but not across days since inoculation (H = 1.34, *P* = 0.97) (Fig. 2C). Pairwise Mann-Whitney *U* tests comparing ranked values of *Cq*s from samples grouped by surface type highlight that both carpet materials (olefin and polyester) are significantly different, after correcting for multiple comparisons (false discovery rate [FDR]-Benjamini/Hochberg, alpha = 0.005), from all other surfaces, but not from each other (Fig. 2B). Other pairwise, significant differences between materials are summarized in Table S1 in the supplemental material. A clustermap of the *U* statistic from the pairwise comparisons effectively clusters samples by material properties, with rough surfaces clustering away from smooth ones (Fig. 2D).

10.1128/mSystems.01136-21.4 TABLE S1 Statistically significant differences from pairwise Mann-Whitney *U* tests between ranked values of average *Cq*s from viral gene calls grouped by surface type after correction for multiple comparisons (**, FDR-Benjamin/Hochberg, alpha = 0.005; N.s. = not significant). Download Table S1, DOCX file, 0.01 MB. Copyright © 2021 Salido et al. 2021 Salido et al. https://creativecommons.org/licenses/by/4.0/ This content is distributed under the terms of the Creative Commons Attribution 4.0 International license.

Because RT-qPCR signal intensity for most surfaces was time invariant, time-collapsed linear regression models relating viral spike-in concentration (log_2_ spike-in) to average *Cq* act as standard curves for estimating viral load on different monitored surfaces from *Cq*. After segregating samples based on the qualitative material categories of smooth or rough, linear regressions aggregating all time points yielded one standard curve for smooth surfaces (*m* = −0.77, *b* = 40.41, *r* = −0.93) (Fig. 2E) and another for rough surfaces (*m* = −0.52, *b* = 39.90, *r* = −0.84) (Fig. 2F). The reduced slope of the latter curve stems from higher loss of spiked-in viral signal to the rough surface matrix.

To ensure that viral signal stability was not a consequence of selection for resilient viral particles through heat inactivation, we repeated a subset of experiments using infectious virus (untreated) in a BSL-3 laboratory using the B.1.351/Beta variant of SARS-CoV-2 originally identified in South Africa. Due to space limitations in the BSL-3 facility, the untreated virus experiment only included two surface types, acrylic and carpet (olefin) but used the same dilution series and sampling plan.

Results from untreated and heat-inactivated virus are concordant. Untreated virus samples cluster with respect to surface type rather than virion status (heat inactivated or untreated) (Fig. 2D). When evaluating acrylic and carpet (olefin) samples alone, a Kruskal-Wallis *H* test shows significant differences in the means of *Cq*s across all groups when samples are grouped by surface type (*H* = 16.37, *P* = 0.00095) (see Fig. S1A in the supplemental material), but not when grouped by virion status (*H* = 1.96, *P* = 0.161) (Fig. S1B). Furthermore, linear regression on *Cq* from paired samples between the heat-inactivated and untreated virus experiments show nearly exact correlation despite the use of different variants (*m* = 1.05, *r* = 0.97) (Fig. S1C).

10.1128/mSystems.01136-21.2 FIG S1 (A) Swarm plot showing distribution of average *Cq*s of viral gene calls for acrylic and carpet (olefin) surfaces for both heat-inactivated and untreated samples. (B) Swarm plot comparing distribution of average *Cq*s of viral gene calls for heat-inactivated or untreated samples. (C) Linear regression on *Cq*s from paired samples between heat-inactivated and untreated samples. Download FIG S1, TIF file, 5 MB. Copyright © 2021 Salido et al. 2021 Salido et al. https://creativecommons.org/licenses/by/4.0/ This content is distributed under the terms of the Creative Commons Attribution 4.0 International license.

### Discussion.

We show that detecting SARS-CoV-2 RNA on indoor surfaces in environments potentially exposed to COVID-19-infected individuals is effective across a variety of surfaces and a range of initial viral loads. Our swabbing and RT-qPCR methods have greater sensitivity from smooth surfaces (such as MFP—commonly found on desktops—or vinyl flooring) than rough surfaces (carpet). The stability of the viral signal across time limits the ability to estimate when the surface was inoculated but demonstrates that signal can be detected a week postexposure. There is a possibility that viral signal could decay over a longer period of time, but because the motivation behind this study was to improve temporal resolution over shorter periods, this was beyond the scope of the present work. To improve temporal resolution, surfaces swabbed for environmental monitoring should be cleaned with soap and water, following CDC recommendations (13), in order to remove viral signals (12). Previous work with comparable methods for SARS-CoV-2 detection from surfaces demonstrated that washing contaminated objects with household dishwashing detergent for ≥1 min removed enough viral RNA traces so that only 20% of the severely contaminated objects had detectable viral RNA. Furthermore, the average viral load of the washed surfaces was reduced by ∼2.5 *Cq*s in comparison to untreated objects (14). Thus, cleaning monitored surfaces with soap and water improves the probability of distinction between persistent or separate exposures in subsequent SARS-CoV-2 detection events.

Although direct inoculation of surfaces with viral particles does not represent interaction with an infected individual in a real-world scenario, we do directly show that untreated and heat-inactivated SARS-CoV-2 particles have similar detectability and stability across surface types. These findings allow the use of heat-inactivated particles in testing and validating environmental monitoring methods and remove the burden of performing such experiments in BSL-3 laboratories.

10.1128/mSystems.01136-21.1 TEXT S1 Materials and Methods. Description of materials and methods for experimental design and sample processing. Download Text S1, DOCX file, 0.02 MB. Copyright © 2021 Salido et al. 2021 Salido et al. https://creativecommons.org/licenses/by/4.0/ This content is distributed under the terms of the Creative Commons Attribution 4.0 International license.

10.1128/mSystems.01136-21.3 FIG S2 Line plots showing the average *Cq*s of RT-qPCR viral signals for positive samples (circles) over 7 days with overlaid scatterplots showing *Cq*s for inconclusive samples (diamonds). Inconclusive samples increase sensitivity of viral detection through surface swabs, seen as increased data points for low viral spike-ins in comparison to positive samples alone. Viral spike-in concentrations reported as GEs from ddPCR. Download FIG S2, TIF file, 6.6 MB. Copyright © 2021 Salido et al. 2021 Salido et al. https://creativecommons.org/licenses/by/4.0/ This content is distributed under the terms of the Creative Commons Attribution 4.0 International license.

10.1128/mSystems.01136-21.5 TABLE S2 Primer and probe sequences used for digital droplet PCR quantification of viral genome equivalents. Download Table S2, DOCX file, 0.01 MB. Copyright © 2021 Salido et al. 2021 Salido et al. https://creativecommons.org/licenses/by/4.0/ This content is distributed under the terms of the Creative Commons Attribution 4.0 International license.

10.1128/mSystems.01136-21.6 TABLE S3 Individual SARS-CoV-2 target gene-positive criteria. Positive results were called on individual SARS-CoV-2 target genes that had a *Cq* of <37 with a confidence of >0.7. A positive result on the extraction control gene (MS2) was called when it had a *Cq* of <37 with a confidence of >0.3. Download Table S3, DOCX file, 0.01 MB. Copyright © 2021 Salido et al. 2021 Salido et al. https://creativecommons.org/licenses/by/4.0/ This content is distributed under the terms of the Creative Commons Attribution 4.0 International license.

10.1128/mSystems.01136-21.7 TABLE S4 RT-qPCR test result-positive criteria. A positive status was called for samples that had at least 2/3 positive calls on the SARS-CoV-2 target genes. An inconclusive status was called on samples with only 1/3 positive calls on the SARS-CoV-2 target genes. A SARS-CoV-2 detected result was called on samples where at least one out of three SARS-CoV-2 targets had a positive call; both positive and inconclusive status samples yielded SARS-CoV-2 detected results. A negative status was called on samples that had a positive call on the control gene and no positive calls on the SARS-CoV-2 target genes. An invalid result was called when neither the control gene nor the viral target genes generated a positive call. Download Table S4, DOCX file, 0.01 MB. Copyright © 2021 Salido et al. 2021 Salido et al. https://creativecommons.org/licenses/by/4.0/ This content is distributed under the terms of the Creative Commons Attribution 4.0 International license.

## Supplementary Material

Reviewer comments

## References

[1] Meyerowitz EA, Richterman A, Gandhi RT, Sax PE. 2021. Transmission of SARS-CoV-2: a review of viral, host, and environmental factors. Ann Intern Med 174:1037. doi:10.7326/L21-0166. PMC7505025 32941052

[2] Parker CW, Singh N, Tighe S, Blachowicz A, Wood JM, Seuylemezian A, Vaishampayan P, Urbaniak C, Hendrickson R, Laaguiby P, Clark K, Clement BG, O’Hara NB, Couto-Rodriguez M, Bezdan D, Mason CE, Venkateswaran K. 2020. End-to-end protocol for the detection of SARS-CoV-2 from built environments. mSystems 5:e00771-20. doi:10.1128/mSystems.00771-20. 33024053 PMC7542562

[3] van Doremalen N, Bushmaker T, Morris DH, Holbrook MG, Gamble A, Williamson BN, Tamin A, Harcourt JL, Thornburg NJ, Gerber SI, Lloyd-Smith JO, de Wit E, Munster VJ. 2020. Aerosol and surface stability of SARS-CoV-2 as compared with SARS-CoV-1. N Engl J Med 382:1564–1567. doi:10.1056/NEJMc2004973. 32182409 PMC7121658

[4] Chin AWH, Chu JTS, Perera MRA, Hui KPY, Yen H-L, Chan MCW, Peiris M, Poon LLM. 2020. Stability of SARS-CoV-2 in different environmental conditions. Lancet Microbe 1:e10. doi:10.1016/S2666-5247(20)30003-3. 32835322 PMC7214863

[5] Harbourt DE, Haddow AD, Piper AE, Bloomfield H, Kearney BJ, Fetterer D, Gibson K, Minogue T. 2020. Modeling the stability of severe acute respiratory syndrome coronavirus 2 (SARS-CoV-2) on skin, currency, and clothing. PLoS Negl Trop Dis 14:e0008831. doi:10.1371/journal.pntd.0008831. 33166294 PMC7676723

[6] Renninger N, Nastasi N, Bope A, Cochran SJ, Haines SR, Balasubrahmaniam N, Stuart K, Bivins A, Bibby K, Hull NM, Dannemiller KC. 2021. Indoor dust as a matrix for surveillance of COVID-19. mSystems 6:e01350-20. doi:10.1128/mSystems.01350-20. 33850045 PMC8547012

[7] Ye G, Lin H, Chen S, Wang S, Zeng Z, Wang W, Zhang S, Rebmann T, Li Y, Pan Z, Yang Z, Wang Y, Wang F, Qian Z, Wang X. 2020. Environmental contamination of SARS-CoV-2 in healthcare premises. J Infect 81:e1–e5. doi:10.1016/j.jinf.2020.04.034. PMC7192102 32360881

[8] Ben-Shmuel A, Brosh-Nissimov T, Glinert I, Bar-David E, Sittner A, Poni R, Cohen R, Achdout H, Tamir H, Yahalom-Ronen Y, Politi B, Melamed S, Vitner E, Cherry L, Israeli O, Beth-Din A, Paran N, Israely T, Yitzhaki S, Levy H, Weiss S. 2020. Detection and infectivity potential of severe acute respiratory syndrome coronavirus 2 (SARS-CoV-2) environmental contamination in isolation units and quarantine facilities. Clin Microbiol Infect 26:1658–1662. doi:10.1016/j.cmi.2020.09.004. 32919072 PMC7481174

[9] Jiang FC, Jiang XL, Wang ZG, Meng ZH, Shao SF, Anderson BD, Ma MJ. 2020. Detection of severe acute respiratory syndrome coronavirus 2 RNA on surfaces in quarantine rooms. Emerg Infect Dis 26:2162–2164. doi:10.3201/eid2609.201435. PMC7454114 32421495

[10] Marotz C, Belda-Ferre P, Ali F, Das P, Huang S, Cantrell K, Jiang L, Martino C, Diner RE, Rahman G, McDonald D, Armstrong G, Kodera S, Donato S, Ecklu-Mensah G, Gottel N, Salas Garcia MC, Chiang LY, Salido RA, Shaffer JP, Bryant MK, Sanders K, Humphrey G, Ackermann G, Haiminen N, Beck KL, Kim HC, Carrieri AP, Parida L, Vázquez-Baeza Y, Torriani FJ, Knight R, Gilbert J, Sweeney DA, Allard SM. 2021. SARS-CoV-2 detection status associates with bacterial community composition in patients and the hospital environment. Microbiome 9:132. doi:10.1186/s40168-021-01083-0. 34103074 PMC8186369

[11] Centers for Disease Control and Prevention. Coronavirus (COVID-19) frequently asked questions. Centers for Disease Control and Prevention, Atlanta, GA. https://www.cdc.gov/coronavirus/2019-ncov/faq.html#Spread. Accessed 1 July 2021.

[12] Centers for Disease Control and Prevention. 2021. SARS-CoV-2 and surface (fomite) transmission for indoor community environments. Centers for Disease Control and Prevention, Atlanta, GA. https://www.cdc.gov/coronavirus/2019-ncov/more/science-and-research/surface-transmission.html. Accessed 1 July 2021. 34009771

[13] Centers for Disease Control and Prevention. 2021. Cleaning and disinfecting your home. Centers for Disease Control and Prevention, Atlanta, GA. https://www.cdc.gov/coronavirus/2019-ncov/prevent-getting-sick/disinfecting-your-home.html. Accessed 10 September 2021.

[14] Salido RA, Morgan SC, Rojas MI, Magallanes CG, Marotz C, DeHoff P, Belda-Ferre P, Aigner S, Kado DM, Yeo GW, Gilbert JA, Laurent L, Rohwer F, Knight R. 2020. Handwashing and detergent treatment greatly reduce SARS-CoV-2 viral load on Halloween candy handled by COVID-19 patients. mSystems 5:e01074-20. doi:10.1128/mSystems.01074-20. 33127739 PMC7743156

