# Supplementary material for: Analysis of SARS-CoV-2 RNA Persistence across Indoor Surface Materials Reveals Best Practices for Environmental Monitoring Programs
Source: mSystems. 2021 Nov 2;6(6):e01136-21. doi: 10.1128/mSystems.01136-21 (PMC8562474; doi:10.1128/mSystems.01136-21)
Supplement: TABLE S1 [file msystems.01136-21-st001.docx]

*Table S1: Statistically significant pairwise comparisons.*

| **Surface Type** | **carpet (olefin)[live]** | **carpet (olefin)** | **carpet (polyester)** | **ceramic tile** | **painted drywall** | **steel** | **vinyl** | **MDF** | **acrylic [live]** | **acrylic** | **glass** |
| --- | --- | --- | --- | --- | --- | --- | --- | --- | --- | --- | --- |
| **carpet (olefin)** | N.s. |  |  |  |  |  |  |  |  |  |  |
| **carpet (olefin)[live]** | N.s. | N.s. |  |  |  |  |  |  |  |  |  |
| **carpet (polyester)** | N.s. | N.s. | N.s. |  |  |  |  |  |  |  |  |
| **ceramic tile** | ** | ** | ** | N.s. |  |  |  |  |  |  |  |
| **painted drywall** | ** | ** | ** | N.s. | N.s. |  |  |  |  |  |  |
| **steel** | ** | ** | ** | ** | ** | N.s. |  |  |  |  |  |
| **vinyl** | ** | ** | ** | N.s. | N.s. | N.s. | N.s. |  |  |  |  |
| **MDF** | ** | ** | ** | N.s. | ** | N.s. | N.s. | N.s. |  |  |  |
| **acrylic [live]** | ** | ** | ** | N.s. | ** | N.s. | N.s. | N.s. | N.s. |  |  |
| **acrylic** | ** | ** | ** | N.s. | ** | N.s. | N.s. | N.s. | N.s. | N.s. |  |
| **glass** | N.s. | ** | ** | N.s. | N.s. | N.s. | N.s. | N.s. | N.s. | N.s. | N.s. |
