# Supplementary figures and images for: Analysis of SARS-CoV-2 RNA Persistence across Indoor Surface Materials Reveals Best Practices for Environmental Monitoring Programs
Source: mSystems. 2021 Nov 2;6(6):e01136-21. doi: 10.1128/mSystems.01136-21 (PMC8562474; doi:10.1128/mSystems.01136-21)

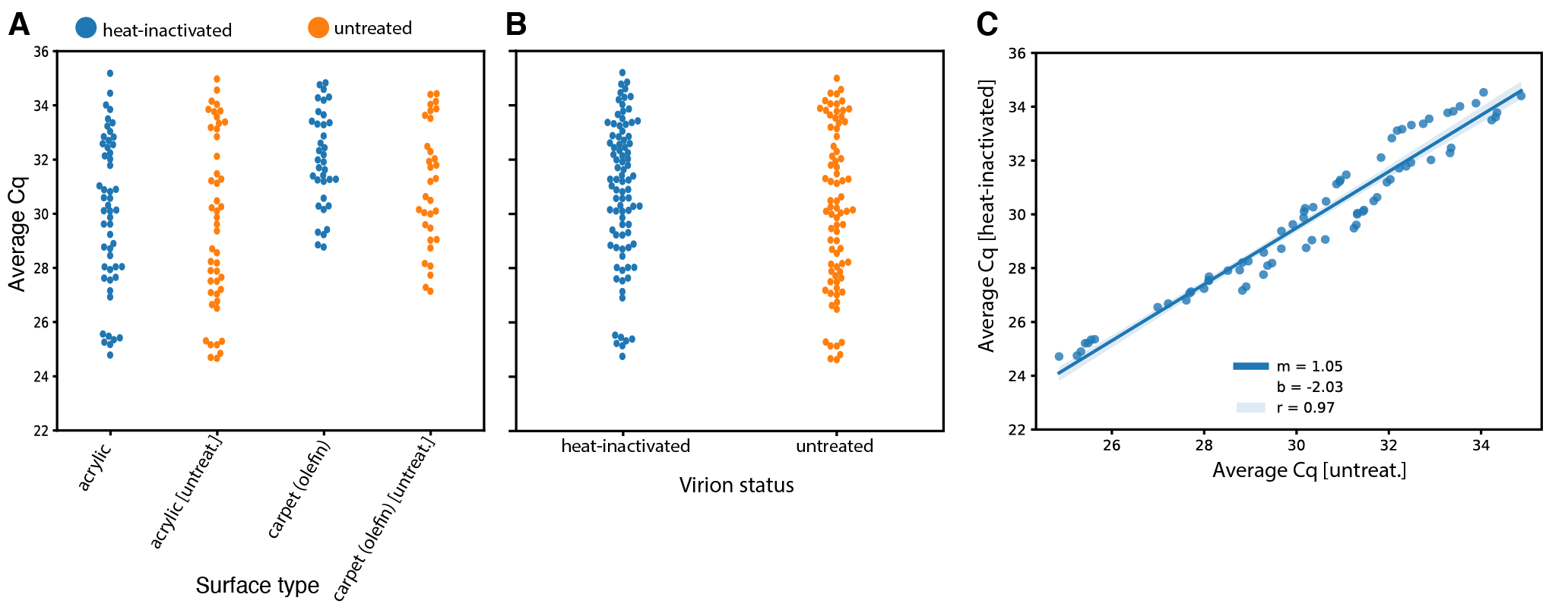

Supplement: FIG S1 [file msystems.01136-21-sf001.tif]

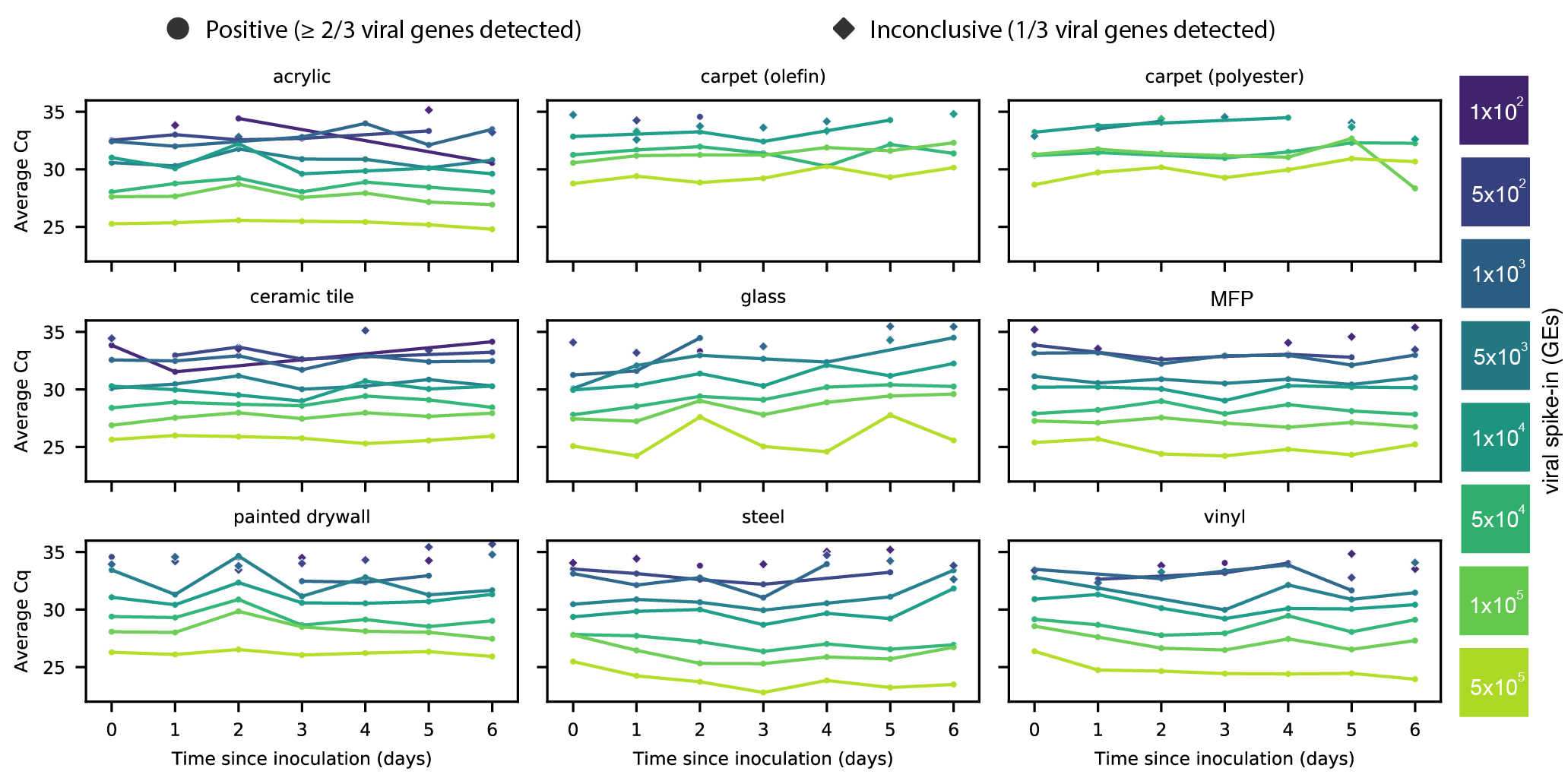

Supplement: FIG S2 [file msystems.01136-21-sf002.tif]
