# Supplementary material for: Analysis of SARS-CoV-2 RNA Persistence across Indoor Surface Materials Reveals Best Practices for Environmental Monitoring Programs
Source: mSystems. 2021 Nov 2;6(6):e01136-21. doi: 10.1128/mSystems.01136-21 (PMC8562474; doi:10.1128/mSystems.01136-21)
Supplement: TEXT S1 [file msystems.01136-21-s0001.docx]

**Materials and Methods.**

Generation of Viruses

SARS-CoV-2 isolates USA-WA1/2020 (NR-52281) and Beta/B.1.351 hCoV-19/South Africa/KRISP-K005325/2020 (NR-54009) were obtained from BEI Resources. WA1 was propagated on VeroE6 cells. B.1.351 was passaged one time through primary bronchial epithelial cells differentiated at air-liquid interface (described below) to select against furin site mutations and then expanded on TMPRSS2-Vero cells (JCRB1819 established by Takeda, M., Sekisui XenoTech LLC). Viral genome copy number was determined by digital droplet PCR (ddPCR) of SARS-CoV-2 ORF1A performed by the Center for AIDS Research (CFAR) Genomics and Sequencing Core at UCSD. Heat inactivation was performed by incubating the virus at 65°C for 30 min. All work with SARS-CoV-2 was conducted in Biosafety Level-3 conditions at the University of California San Diego.

Bronchial epithelial cell culture and differentiation at air-liquid interface

Primary normal human bronchial epithelial cells (NHBECs) were acquired from Lonza (NHBE CC-2540; Walkersville, MD). The cell passage 1’ frozen vial of NHBECs provided were tested and certified negative for HIV, HBV, HCV, sterility, and mycoplasma. NHBECs were revived from cryopreservation and expanded with PneumaCultTM Ex-Plus media (StemCell, 05040; Tukwila, WA). When cells were ~70-80% confluent, they were dissociated and seeded on collagen-coated transwells (Corning, 29442-082) pre-coated with 50 µg/mL Collagen type I from rat tail (BD Biosciences, 354236) at 7.5 µg/cm2. Collagen-coated transwells were rehydrated with 100 µL PneumaCult TM Ex-Plus media at 37°C, 5% CO_2_ for 30 minutes prior to seeding NHBECs for ALI culture. NHBECs were then seeded at 50,000 cells in 200 µL of media per collagen-coated transwell with 500 µL in the basolateral chamber. Media was changed on days 1 and 3 and then exchanged for PneumaCult TM ALI media (StemCell, 05021) supplemented with 10 μM ROCK inhibitor (Tocris, Y-27632) on day 4-7. Upon reaching confluency, approximately on Day 8, the apical media was removed, and the basal media replaced with PneumaCult TM ALI media without Y-27632. On the following day, the basal media was changed with fresh ALI media. Subsequent media changes were every 2-3 days. On Day 14 post-airlift, the apical surfaces were washed with DPBS, once per week. Cells were grown in 37°C, 5% CO_2_ incubator until four weeks airlifted.

Infection of NHBECs at ALI

Prior to infection, apical chamber was incubated with two 30 min washes of PBS at 37°C, 5% CO2. Virus diluted in PBS was added to the apical chamber in 100uL and removed after 24h. Apical washes (150uL PBS with 10 min incubation at 37°C, 5% CO2,) were taken daily and stored at -80°C. Titer was determined by focus forming assay on TMPRSS2-Vero cells.

Digital droplet PCR

RNA was extracted using the QIAamp Viral RNA Mini kit (QIAGEN) following manufacturer’s instructions. Equal volumes of RNA were run in triplicate and amplified in a 20 µL reaction (One-step RT-ddPCR Advanced Kit; Bio-Rad) with 900nM each primer and 250nM probe_FAM/ZEN/IBFQ ORF1a with RNAse P as an internal control. Droplets were generated (QX200 droplet generator; Bio-Rad) and placed in ddPCR™ 96-Well Plates (Bio-Rad). Plates were heat-sealed and transferred to an Applied Biosystems Veriti 96 Well Thermal Cycler. PCR reaction conditions were 25°C for 3 min, 45°C for 60 min, 95°C for 10 min, and then cycled 45 times at 95°C for 30 sec, 60°C for 60 sec. Droplets were read on a QX200 droplet reader (Bio-Rad). The primers and probes use are described in Table S2.

Preparing Surfaces

The acrylic (Plaskolite, MC-05), carpets (olefin (TrafficMaster, 0701649510) and polyester (TrafficMaster, 7PD5N480144H)), ceramic tile (Daltile, MA031014HD1P2), glass (Gardner Glass Products, 11216), melamine-finished particleboard (MFP) (Roseburg, 371595), steel (M-D Building Products, 56020), and vinyl flooring (Lifeproof, I536111L) were purchased from a hardware store and used without modification. A slab of drywall (USG Sheetrock Brand, 141133) was painted with three coats of a white, matte interior paint and primer (Glidden, GLN9012N) and allowed to dry. Each of the nine surfaces were divided into 5 cm by 5 cm grids, sprayed with 70% v/v ethanol in water, and wiped down with a paper towel. The grid sections on the carpet materials were separated by 1 cm to prevent diffusion of the viral inoculum into neighboring sections.

Inoculating Surfaces

In both the heat-inactivated and untreated virus experiments, a concentrated stock of SARS-CoV-2 was diluted to create solutions with concentrations of 5x10^4^, 1x10^4^, 5x10^3^, 1x10^3^, 5x10^2^, 1x10^2^, 5x10^1^, 1x10^1^ GEs/µL. A 10 μL volume of each solution was slowly dispensed onto a grid section while moving the pipette tip in a raster pattern in an attempt to evenly distribute the aliquot over each grid section’s 25 cm^2^ area. Water was dispensed in the same fashion onto sections that were swabbed each day as negative controls. The grid sections sampled for the first timepoint were allowed to dry for 1 hr before swabbing.

The surfaces inoculated with untreated virus were placed in tupperware containers in between inoculation and swabbing so that they could be safely stored outside of a biosafety cabinet. Surfaces inoculated with heat-inactivated virus were kept on designated bench tops without a container.

Swabbing

For each sample, a 1 mL sample collection tube (ThermoFisher Scientific, 3740TS) containing 800 µL of 0.5% w/v sodium dodecyl sulfate (SDS) (Acros Organics, 230420025) in water was prepared. This solution was chosen because 0.5% SDS has been shown to effectively inactivate amounts of SARS-CoV-2 used in this study after 30 minutes of contact time [**1, 2**]. To recover viral genetic material from the surface sections, a flocked swab (Affordable IHC Solutions) was pre-moistened with 0.5% SDS solution from a 1mL sample collection tube and the grid section was swabbed in a horizontal zig zag pattern, followed by a vertical zig zag pattern and finally, the same pattern in a diagonal orientation. The swab was rotated while swabbing and sufficient pressure was applied to maximize the contact surface area between the flocculated swab and the surface being swabbed. The flocculated end of the swab was placed back into the same sample collection tube and the swab shaft was broken at the designated break point (3 cm break point). The 1D barcode of each tube was scanned using a handheld barcode scanner in order to link the sample to the experimental conditions of the swabbed surface.

Sample accession

Sample tubes were randomized and racked in barcoded 96-well tube racks (ThermoFisher Scientific, 4897). Sample identity was tracked using 2D barcodes at the bottom of each tube, which were scanned using a VisionMate Barcode Reader (ThermoFisher Scientific, 312800). A laboratory information management system (LIMS) was employed to track samples in multiwell plate formats using plate barcodes. [**3,4**]

Nucleic acid extraction:

Individual 96-well tube racks were vortexed for 5 minutes to promote the suspension of viral particles from the swabs into the 0.5% SDS solution. Afterwards, 150 µL of the suspension buffer (0.5% SDS) were transferred with a multichannel pipette into barcoded deep well extraction plates and processed using the Omega MagBind Viral DNA/RNA kit (Omega Bio-Tek, M6246) on the Kingfisher Flex (ThermoFisher Scientific) platform following manufacturer’s protocol with the following modifications: only 150µL of sample input was used (instead of the recommended 200µL) and 10 µL of MS2 phage was added to each well as an extraction control.

RT-qPCR (Multiplexed TaqPath)

Viral gene detection assays were performed using the RT-qPCR-based TaqPath™ COVID-19 Combo Kit (ThermoFisher Scientific, A47814) on a QuantStudio 7 Pro with a 384-well sample block (ThermoFisher Scientific) according to the manufacturer’s protocol with the following modifications: 2 µL of purified RNA was added to a 1 µL reaction mix containing 0.75 µL TaqPath 4x Enzyme mix (ThermoFisher Scientific, A28523), 0.15 µL multiplex probe mix, and 0.1 µL nuclease free water, for a total reaction volume of 3 µL. Low volume transfers (< 5µL) were done with Mosquito HV Liquid Handlers (SPT Labtech). The following RT-qPCR cycling conditions were used: 25°C for 2 minutes, 53°C for 10 minutes, 95°C for 2 minutes, 55 cycles of 95°C for 3 seconds, and 60°C for 30 seconds. The signal was measured at the end of each 30 second interval at 60°C. Baseline determination and quantification cycle (Cq) signal determination were made using the Design and Analysis v2.4.3 software (Applied Biosystems) using the relative threshold (Crt) method. Positive calls for individual gene reporters were made according to Table S3.

Because Inconclusive (only ⅓ viral target genes detected) samples allowed for increased sensitivity without considerable introduction of noise (Fig S2), the criteria for SARS-CoV-2 Detected results from surface swabs was defined as at least one out of three viral gene targets amplified. The US Food and Drug Administration has issued Emergency Use Authorization (EUA) for more than 150 RT-qPCR assays for the detection of SARS-CoV-2, the majority of which define a positive result as amplification in a single target [**5**]. The environmental surface swab result criteria used for this study is described in Table S4.

Controls

9 out of 81 (%11.1) blank surface swabs (surfaces inoculated with water) showed some viral signal. Contaminated blank surface swabs were exclusive to carpet (olefin), vinyl, acrylic, and MFP surface types, but not exclusive to any particular timepoint. All RT-qPCR positive controls behaved as expected. 2 out of 21 RT-qPCR negative controls failed to detect the extraction control (MS2 phage), but in both of those occasions, an alternative RT-qPCR negative control within the same RT-qPCR plate, respectively, yielded valid Not Detected results.

References

1. Patterson EI, Prince T, Anderson ER, Casas-Sanchez A, Smith SL, Cansado-Utrilla C, Solomon T, Griffiths MJ, Acosta-Serrano Á, Turtle L, Hughes GL. 2020. Methods of Inactivation of SARS-CoV-2 for Downstream Biological Assays. J Infect Dis 222:1462–1467.

2. Welch SR, Davies KA, Buczkowski H, Hettiarachchi N, Green N, Arnold U, Jones M, Hannah MJ, Evans R, Burton C, Burton JE, Guiver M, Cane PA, Woodford N, Bruce CB, Roberts ADG, Killip MJ. 2020. Analysis of inactivation of SARS-CoV-2 by specimen transport media, nucleic acid extraction reagents, detergents, and fixatives. J Clin Microbiol 58.

3. Morgan SC, Aigner S, Anderson C, Belda-Ferre P, Hoff D, Marotz CA, Sathe S, Zeller M, Ahmed N, Audhya X, Baer NA, Barber T, Barrick B, Batachari L, Betty M, Blue SM, Brainard B, Buckley T, Case J, Chacón M, Cheung W, Chong L, Crescini ES, DeGrand S, Dimmock DP, Joelle Donofrio- J, Eisner ER, Estaki M, Franco Vargas L, Freddock M, Gallant RM, Galmozzi A, Gao NJ, Gilmer S, Grzelak EM, Hakim A, Hart J, Hobbs C, Humphrey G, Ilkenhans N, Jacobs M, Kahn CA, Kapadia BK, Kim M, Kurian S, Lastrella AL, Lawrence ES, Lee K, Liang Q, Liliom H, Sardo L, Logan R, Machnicki M, Magallanes CG, Malacki D, Marina RJ, Marsh C, Martin NK, Matteson NL, Maunder DJ, McBride K, McDonald B, Meadows AR, Meyer M, Morey AL, Mueller JR, Ngo TT, Nguyen J, Nguyen V, Nicholson LJ, Nouri A, Nunez E, Tyler Ostrander R, Pantham P, Park SS, Picone D, Plascencia A, Pratumchai I, Franc Ragsac M, Richardson AC, Robles-Sikisaka R, Ruiz CA, Ryan J, Sacco L, Saraf S, Seaver P, Smoot EW, Sweeney KM, Tekkatte C, Tsai R, Valentine H, Walsh S, Williams A, Yi Wu M, Xia B, Yee B, Zhang JZ, Andersen KG, Farnaes L, Knight R, Laurent LC. 2021. Automated, miniaturized, and scalable screening of healthcare workers, first responders, and students for SARS-CoV-2 in San Diego County. medRxiv 2021.06.25.21257885.

4. Sathe S, Mah C, Ahmed N, Ragsac MF, Williams J, Yeo G. 2020. INSPECT sample tracking system Coronavirus Method Development Community <https://doi.org/10.17504/protocols.io.bis8kehw>.

5. MacKay MJ, Hooker AC, Afshinnekoo E, Salit M, Kelly J, Feldstein J V., Haft N, Schenkel D, Nambi S, Cai Y, Zhang F, Church G, Dai J, Wang CL, Levy S, Huber J, Ji HP, Kriegel A, Wyllie AL, Mason CE. 2020. The COVID-19 XPRIZE and the need for scalable, fast, and widespread testing. Nat Biotechnol. Nature Research.
