# Supplementary material for: Analysis of SARS-CoV-2 RNA Persistence across Indoor Surface Materials Reveals Best Practices for Environmental Monitoring Programs
Source: mSystems. 2021 Nov 2;6(6):e01136-21. doi: 10.1128/mSystems.01136-21 (PMC8562474; doi:10.1128/mSystems.01136-21)
Supplement: TABLE S2 [file msystems.01136-21-st002.docx]

*Table S2. Primer and probe sequences for digital droplet PCR (ddPCR)*

| Primer name | Modification | Sequence (5’-3’) |
| --- | --- | --- |
| COVID19_ORF1a-F |  | GTCGTAGTGGTGAGACACTTG |
| COVID19_ORF1a-R |  | GGCCACCAGCTCCTTTATTA |
| COVID19_ORF1a-Prb | FAM/ZEN/IBFQ | ATACCAGTGGCTTACCGCAAGGTT |
| RPP30-F |  | GATTTGGACCTGCGAGCG |
| RPP30-R |  | GCGGCTGTCTCCACAAGT |
| RPP30-Prb | HEX/ZEN/IBFO | CTGACCTGAAGGCTCT |
