# Supplementary material for: Analysis of SARS-CoV-2 RNA Persistence across Indoor Surface Materials Reveals Best Practices for Environmental Monitoring Programs
Source: mSystems. 2021 Nov 2;6(6):e01136-21. doi: 10.1128/mSystems.01136-21 (PMC8562474; doi:10.1128/mSystems.01136-21)
Supplement: TABLE S3 [file msystems.01136-21-st003.docx]

*Table S3. Individual SARS-CoV-2 target gene positive criteria.*

| **Individual Target Passing Criteria** | | |
| --- | --- | --- |
| **Target** | **Cq Range** | **Cq Confidence** |
| ORF1ab (viral) | <37 | >0.7 |
| N Gene (viral) | <37 | >0.7 |
| S Gene (viral) | <37 | >0.7 |
| MS2 (control) | <37 | >0.3 |
