# Supplementary material for: Analysis of SARS-CoV-2 RNA Persistence across Indoor Surface Materials Reveals Best Practices for Environmental Monitoring Programs
Source: mSystems. 2021 Nov 2;6(6):e01136-21. doi: 10.1128/mSystems.01136-21 (PMC8562474; doi:10.1128/mSystems.01136-21)
Supplement: TABLE S4 [file msystems.01136-21-st004.docx]

*Table S4. Environmental Surface Swab RT-qPCR test result criteria.*

| **ORF1ab** | **N Gene** | **S Gene** | **MS2** | **Status** | **Result** |
| --- | --- | --- | --- | --- | --- |
| Neg | Neg | Neg | Neg | Invalid | Invalid |
| Neg | Neg | Neg | Pos | Negative | SARS-CoV-2 Not Detected |
| Only one target=Pos | | | Pos/Neg | Inconclusive | SARS-CoV-2 Detected |
| Two or more Targets=Pos | | | Pos/Neg | Positive | SARS-CoV-2 Detected |
